# Feline intralenticular *Encephalitozoon cuniculi*: three cases from California

**DOI:** 10.1177/20551169221106721

**Published:** 2022-08-02

**Authors:** Joie Lin, Barbara Nell, Taemi Horikawa, Mitzi Zarfoss

**Affiliations:** 1 School of Veterinary Medicine, University of California-Davis, Davis, CA, USA; 2 Department for Small Animals and Horses, Veterinary University of Vienna, Vienna, Austria; 3 Ophthalmology for Animals, Aptos, CA, USA; 4 Pets Referral Center, Berkeley, CA, USA

**Keywords:** *Encephalitozoon cuniculi*, uveitis, cataracts, phacoemulsification

## Abstract

**Case series summary:**

Three domestic shorthair cats from California presented to veterinary ophthalmologists with immature cataracts. Other presenting clinical signs included corneal edema, anisocoria, anterior uveitis, elevated intraocular pressure, blepharospasm and/or lethargy. All patients were immunocompromised due to concurrent diseases and/or immunomodulatory drugs. Diagnostics included serial comprehensive ophthalmic examinations with tonometry, ocular ultrasound, electroretinogram and testing for other causes of feline uveitis. Testing for *Encephalitozoon cuniculi* included serology, histopathology and/or PCR of aqueous humor, lens material or paraffin-embedded whole eye. Treatments included antiparasitic medication, anti-inflammatory medication and supportive care in all three cases. Surgical treatment included enucleation (one case), bilateral phacoemulsification and unilateral intraocular lens placement (one case) and bilateral phacoemulsification with bilateral endolaser ciliary body ablation and bilateral intraocular lens implantation (one case). Both cats for which serologic testing for *E cuniculi* was performed were positive (1:64–1:4096). In all cats, diagnosis of intraocular *E cuniculi* was based on at least one of the following: lens histopathology or PCR of aqueous humor, lens material or paraffin-embedded ocular tissue. The clinical visual outcome was best in the patient undergoing phacoemulsification at the earliest stage of the cataract.

**Relevance and novel information:**

*Encephalitozoon cuniculi* should be considered as a differential cause of cataracts and uveitis in cats in California, the rest of the USA and likely worldwide.

## Introduction

*Encephalitozoon cuniculi* is a worldwide microsporidian.^1,2^ Spores can be transmitted via respiratory, oral,^3^ conjunctival,^4^ intranasal, intraovarial or transplacental routes.^5^ In veterinary ophthalmology, *E cuniculi* is predominantly known as a cause of cataracts and phacoclastic uveitis in rabbits;^6,7^ after transplacental transmission, spores are speculated to enter the lens via the lenticular blood supply while it is still present.^8^ However, a recent study with immunohistochemical evidence of intralenticular *E cuniculi* after oral infection in 4-month-old, immunocompetent, specific pathogen-free rabbits^9^ suggested that alternative mechanisms for lens infections are possible. Reports of ocular involvement in other species are limited and include cataract and uveitis in a snow leopard in France,^10^ cataract, uveitis and chorioretinal lesions in dogs in Europe,^11^ keratitis and uveitis in an American cat,^12^ polyarteritis nodosa and cataract in a blue fox,^13^ cataract and neurologic lesions in mink in Norway^14^ and keratoconjunctivitis in an American cockatoo.^15^ In addition, *E cuniculi* has been thoroughly investigated as a cause of feline cataract in Austria.^16^ This report includes the clinical data from three feline cases of intralenticular *E cuniculi* in California, USA; one case is discussed at length (case 1), while the other two cases (cases 2 and 3) are summarized in Table 1.

**Table 1:** Case summaries

	Case 1	Case 2	Case 3
Signalment	3-year-old MN DSH	15-year-old FS DSH	1.75-year-old FS DSH
Presenting clinical signs	Blepharospasm, anisocoria, lethargy	Rapid-onset cataracts, 6 months after diagnosis of intestinal lymphoma	Upper respiratory signs, ocular discharge, cloudy opacity OS
Description of initial cataract	Focal anterior cortical cataracts OU (see Figure 1)	Immature cataracts OU	Incipient peripheral cortical cataracts OU (see Figure 2)
Degree of uveitis at presentation	Mild corneal edema OU, moderate keratic precipitates OU, aqueous flare OU (trace OD and 1/4+ OS)	No flare, rubeosis or episcleral injection OU	• OD: no aqueous flare, mild keratic precipitates • OS: rubeosis iridis, 3–4/4+ aqueous flare, keratic precipitates
IOP, lowest to highest	• 16–37 mmHg OD • 8–44 mmHg OS	• 9–70 mmHg OD • 3–56 mmHg OS	• 11–30 mmHg OD • 15–62 mmHg OS
Systemic testing: negative, normal results	• CBC and serum chemistry • Seronegative: *T gondii* IgG/IgM, FIV, FeLV antigen (IDEXX Reference Laboratory) • Upper respiratory PCR panel (IDEXX Reference Laboratory): *C felis*, feline calicivirus, *M felis* and influenza A • Thoracic radiographs • Aqueous humor PCR was negative: FHV-1, FCoV, FeLV, *Bartonella species, C neoformans, T gondii*, FIV • Normal cytology of a mesenteric lymph node • FIP PCR (blood): negative	• Seronegative: FeLV/FIV/*T gondii* • FCoV IFA <1:400	• Seronegative: FIV, coronavirus (*T gondii*), *C neoformans* (Antech Diagnostics)
Systemic testing positive results	• FCoV titer 1:3200 • *C neoformans* titer 1:8 • *B henselae* and *B clarridgeiae* titer =1:128 • Fecal testing positive: *Giardia* (ELISA), *T foetus*, *Cryptosporidium* species, *Giardia* species, FCoV and *C perfringens* alpha toxin gene (PCR; IDEXX Reference Laboratory) • Upper respiratory PCR panel, FHV-1 positive at 0.160 thousands/swab (latent infection; IDEXX Reference Laboratory) • Abdominal ultrasound (splenomegaly and mild mesenteric lymphadenopathy) • Repeat coronavirus titer 1:1600	NA	• *Bartonella* species = 4+ strong positive (Western blot, National Veterinary Laboratory) • FeLV-positive (Antech Diagnostics)
Systemic diagnoses	• FCoV • *Bartonella* species serologic positive • Cryptococcosis • FHV-1 • Giardiasis • *T foetus* infection • Cryptosporidiosis	• Intestinal lymphoma • FHV-1 (suspected, not confirmed)	• FeLV *• Bartonella* species serologic positive
Medical treatment for *E cuniculi*	Fenbendazole 50 mg/kg PO q24h for 3 weeks, repeated twice	Fenbendazole 70 mg/kg PO q24h for 3 weeks	Fenbendazole 50 mg/kg PO q24h for 10 days (multiple courses)
Surgical treatment	• Phacoemulsification OU • Intraocular lens OS	• Phacoemulsification OU • Intraocular lens OU • Endoscopic cyclophotocoagulation OU	• Enucleation OS
Glaucoma treatment	• 2% dorzolamide/0.5% timolol OU q12h • 2% dorzolamide OU q8h	• 2% dorzolamide OU q12h–q6h • Methazolamide 7.5 mg PO q24h • 0.5% timolol OU q12h	• Methazolamide (Wedgewood Compounding Pharmacy) 15 mg PO q24h–q12h
Uveitis treatment	• Onsior (robenacoxib; Elanco) • Diclofenac 0.1% ophthalmic solution (Bausch and Lomb) OU q12h • Topical 1% prednisolone acetate suspension (Pacific Pharma) OU q24h–q12h • 0.1% nepafenac ophthalmic suspension (Nevanac Alcon) OU q24h–q6h	• Neomycin polymyxin B sulfates and dexamethasone OU three times weekly • 1% prednisolone acetate OU q8h	• Dexamethasone 0.1% (Bausch and Lomb) OS q24h–q12h
Keratitis treatment	• Bacitracin neomycin gentamicin ophthalmic ointment (AC Pharmaceuticals) OU q8h • Famciclovir (Neogen) 250 mg PO q6h • Ofloxacin 0.3% OU q24h • Optixcare (Optixcare Eye Lube Plus; Aventix) OU q12h • 0.5% cidofovir (Wedgewood Compounding Pharmacy) OU q12h	• 0.5% cidofovir OU q12h • Famciclovir 125 mg PO q12h-q8h • Remend corneal repair gel (Elanco) OU q8h • Ofloxacin 0.3% OU q6h • 5% NaCl ophthalmic ointment OU q6h • Autologous serum OU q6h • 2% ciclosporin aqueous (for stromal keratitis; Stokes Compounding Pharmacy) OU q24h • Buprenorphine 0.005–0.01 mg/kg transbuccal q8h	NA
Medical treatment for other conditions	• Fluconazole 10.7 mg/kg PO q12h • Doxycycline 4.3 mg/kg PO q12h for 3 weeks • Ronidazole (for diarrhea) • Buprenorphine (0.5 mg/ml; Hikma)	• Prednisolone 5 mg PO q24h • Chlorambucil 2 mg PO q12h for four doses q2weeks • Vitamin B12/cobalamin 250 µg monthly • SC fluids for hyporexia	• Doxycycline (Road Runner Compounding Pharmacy) 6 mg/kg PO q12h for 25 days (multiple courses) • Oral pradofloxacin (Veraflox; Elanco) • Prednisolone 1 mg PO EOD
Duration of follow-up	1.4 years post-phacoemulsification	1 year post-phacoemulsification	6 years after initial examination, 5.5 years post-enucleation OS
Outcome	• OS: pseudophakic • OD: aphakic • OU: menace and PLR positive, comfortable, no aqueous flare, mild capsular opacity, numerous, punctate, gray, slightly hyporeflective retinal lesions • IOP 18/17 mmHg OD/OS	• OU: pseudophakic, menace negative but patient navigated the room well, PLR and dazzle positive, comfortable, no flare, mild retinal degeneration • IOP 24/25 mmHg OD/OS	• OS: enucleation • OD: mature cataract menace negative, positive PLR and dazzle, mild keratic precipitates and rubeosis iridis, no aqueous flare, fluorescein negative • IOP 21 mmHg

MN = male neutered; DSH = domestic shorthair; FS = female spayed; IOP = intraocular pressure; CBC = complete blood count; *T gondii* = *Toxoplasma gondii*; FIV = feline immunodeficiency virus; FeLV = feline leukemia virus; *C felis* = *Chlamydophila felis; M felis* = *Mycoplasma felis*; FHV-1 = feline herpesvirus-1; FCoV = feline coronavirus; *C neoformans* = *Cryptococcus neoformans*; FIP = feline infectious peritonitis; IFA = immunofluorescence; *B henselae* = *Bartonella henselae; B clarridgeiae* = *Bartonella clarridgeiae; T foetus* = *Tritrichomonas foetus; C perfringens* = *Clostridium perfringens*; NA = not available; *E cuniculi* = *Encephalitozoon cuniculi*; SC = subcutaneous; EOD = every other day; PLR = pupillary light reflex

## Case series description

### Case 1

A 3-year-old male castrated domestic shorthair cat presented to an emergency service for blepharospasm, anisocoria and lethargy. The patient had a history of chronic upper respiratory infections and diarrhea. It had been rescued from a southern Californian shelter and lived indoors in San Francisco.

On initial emergency examination, the patient’s intraocular pressure was 26 mmHg OD and 33 mmHg OS, with mild corneal edema OU. Treatment included robenacoxib (2 mg/kg SC once [Onsior; Elanco]), ofloxacin (OU q8h) and 2% dorzolamide/0.5% timolol (OU q12h). See Table 1 for the diagnostic testing results (testing for *E cuniculi* was not immediately performed).

Initial ophthalmic examination revealed menace response and pupillary light reflex (PLR) positive OU, mild corneal edema with moderate keratic precipitates OU, aqueous flare OU (trace OD and 1/4+ OS), focal posterior synechiae OS and focal anterior cortical cataracts OU (Keeler PSLClassic). Intraocular pressure was 20 mmHg OD and 12 mmHg OS (Icare Tonovet; Icare Finland). Retinal examination (Keeler Vantage Indirect) was normal except for numerous, punctate, gray, slightly hyporeflective lesions in the dorsal retina OU. Treatment with topical diclofenac 0.1% ophthalmic solution OU q12h (Bausch and Lomb) was initiated. Treatment with fluconazole (10.7 mg/kg PO q12h long-term for *Cryptococcus neoformans*) and doxycycline (4.3 mg/kg PO q12h for 21 days for *Bartonella* species) was initiated.

One week after initial presentation, aqueous flare was unimproved. Treatment with topical 1% prednisolone acetate suspension (OU q12h; Pacific Pharma) and topical 0.5% cidofovir (OU q12h; Wedgewood Compounding Pharmacy) were added.

One month after initial presentation, the patient developed a large superficial corneal ulcer OS, suspected to be related to herpes exacerbated by topical steroids. Prednisolone acetate was discontinued, and bacitracin neomycin gentamicin ophthalmic ointment (AC Pharmaceuticals, Arroyo Grande CA) was added OU q8h.

Approximately 2 months after the initial examination, the corneal ulcer OS persisted. Aqueous flare was trace OD and 1/4+ OS, with an intraocular pressure (IOP) of 19 mmHg OD and 44 mmHg OS. Aqueocentesis was performed OS, and aqueous humor was submitted for PCR to determine if *C neoformans, Bartonella* species or feline coronavirus (FCoV) were the cause of uveitis. Because the patient’s cataracts appeared to be similar to those described in a previous report,^16^ a special request was made to IDEXX to add an *E cuniculi* PCR test. A contact lens (PureVision BC 8.6; Bausch and Lomb) was placed, and a partial lateral temporary tarsorrhaphy was performed for 2 weeks. Medication administered immediately after the procedures included bacitracin neomycin gentamicin ophthalmic ointment (OU q8h) and dorzolamide HCl/timolol maleate (OS q8h; Bausch and Lomb). Oral medications included ronidazole (for diarrhea), buprenorphine (Hikma 0.5 mg/ml), robenacoxib (6 mg PO q24h for 3 days [Onsior; Elanco]) and famciclovir (250 mg PO q12h; Neogen). Aqueous humor cytology (Veterinary Diagnostics) showed increased cellularity, with 68% mixed (mostly mature) lymphocytes, 15% quiescent to vacuolated macrophages and 17% non-degenerate to slightly poorly preserved neutrophils (see Tables 1 and 2). Given the positive aqueous humor PCR result for *E cuniculi*, treatment with fenbendazole (50 mg/kg PO q24h for 3 weeks) was initiated.

**Table 2:** *Encephalitozoon cuniculi* testing

	Case 1	Case 2	Case 3
Serology (IgG)	1:64*	NA	1:4096 (2014) then 1:256 (2018)*
PCR	• Aqueocentesis fluid, positive^†^ • Lens material, positive, strain II^‡^	• Phacoemulsified lens fluid, positive^§^ • Urine negative^¶^	Paraffin scrolls of enucleated eye (OS), positive^¶^
Histopathology	Lens capsule: Gram-positive, Ziehl–Neelsen acid-fast positive^∞^	NA	Globe: intralenticular organisms, Gram-positive, variably acid-fast, Luna stain positive (see Figure 3)^¶^

* University of Miami Avian & Wildlife Laboratory

† IDEXX Reference Laboratories

‡ Department for Pathobiology, Veterinary University Vienna

§ Athens Veterinary Diagnostic Laboratory, University of Georgia

¶ Comparative Pathology Laboratory, University of California, Davis

∞ Comparative Ocular Pathology Laboratory of Wisconsin

NA = not available

Three months after initial presentation, ophthalmic examination indicated similar signs of uveitis with punctate fluorescein positivity OS only, and IOP was 16 mmHg OD and 8 mmHg OS. Topical 0.1% nepafenac ophthalmic suspension (OU q12h; Nevanac Alcon) was initiated.

Four months after initial presentation, aqueous flare had resolved with normal IOP without any dorzolamide/timolol in the previous 3 days. Given the anticipated difficulty in controlling uveitis medically and the likelihood that cataracts would progress in the long term, cataract surgery was considered. Owing to the patient’s positive FCoV status, thoracic radiographs (unremarkable), abdominal ultrasound (splenomegaly and mild mesenteric lymphadenopathy) and ultrasound-guided aspiration of a mesenteric lymph node (cytologically normal) were performed (see also Table 1).

**Figure 1:**
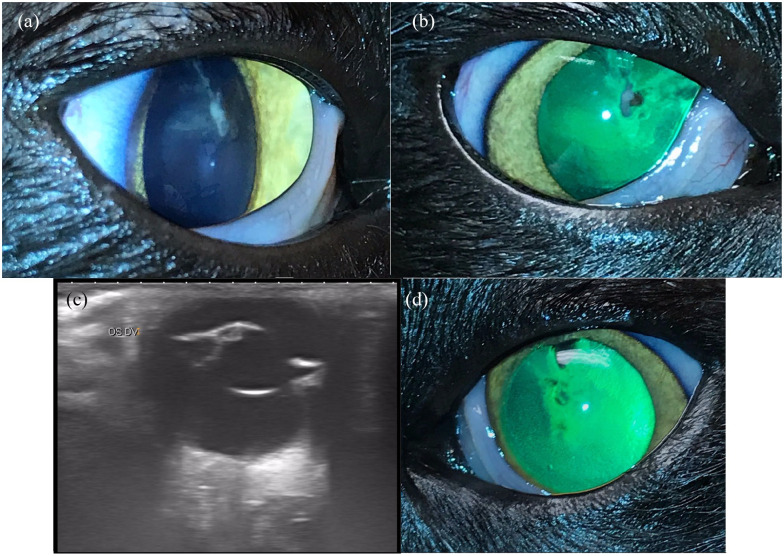
Case 1: (a,b,d) clinical photos and (c) ultrasound image. Pupils were pharmacologically dilated with 1% tropicamide ophthalmic in (a), (b) and (d) (Akorn). (a,b) OD focal anterior subcapsular to anterior cortical cataract and focal pigment on lens capsule. The lens capsule appeared focally wrinkled at the site of the cataract clinically, but no capsular tears were visible on slit-lamp examination. (c) OS: vertical ultrasound image showing echoic dorsal anterior subcapsular cataract with anterior cortical to nuclear extension. The lens capsule was interpreted to be intact via ultrasound. Other than lens abnormalities, anterior and posterior segments were within normal limits. (d) OS: clinical photo showing retro-illumination of focal subcapsular to anterior cortical cataract (dark lenticular opacities). Images courtesy of Dr Mitzi Zarfoss

Five months after initial presentation, *E cuniculi* serology was 1:64 (University of Miami Avian & Wildlife Laboratory; see Table 2 for the full list of *E cuniculi* tests). Electroretinogram (ERG Retinographics BNP200) was normal with b-wave amplitudes >300 µV OU. Ocular ultrasound (Toshiba AplioMX) was normal OU except for multifocal capsular/cortical lens irregularities OU (Figure 1). Phacoemulsification was performed OU (Acrivet Alexos). An intraocular lens was placed OS only (An-lens MC1-13) due to excision of a peripheral capsular plaque necessitating excess capsule removal OD. Immediate postoperative medications included 0.3% ofloxacin ophthalmic solution (OU q6h; Bausch and Lomb), 0.5% cidofovir (OU q12h), 0.1% Nevanac (OU q6h), 1% prednisolone acetate (OU q6h), 2% dorzolamide ophthalmic solution (OU q8h; Micro Labs) and Optixcare (OU q12h; Optixcare Eye Lube Plus Aventix). Oral medications included fenbendazole (50 mg/kg PO q24h for 3 weeks), fluconazole, amoxicillin trihydrate/clavulanate potassium (62.5 mg PO q12h; Zoetis), transmucosal buprenorphine (0.02 mg/kg q8h; Wedgewood Compounding Pharmacy) and robenacoxib (6 mg PO q24h for three doses [Onsior; Elanco]). One day postoperatively, IOP was 37 mmHg OD and 43 mmHg OS but normalized after two extra doses of 2% dorzolamide/0.5% timolol OU. Perincisional superficial corneal ulcers and 1/4+ aqueous flare were present OU.

Lens capsule plaques were submitted to the Comparative Ocular Pathology Laboratory of Wisconsin. Histopathology of the right lens capsule presented moderate numbers of foamy-to-epithelioid macrophages with numerous 1–3 µm, rod-shaped microsporidia consistent with *E cuniculi*. The organisms were strongly Gram positive and lightly Ziehl–Neelsen acid-fast positive (see Table 2).

Postoperatively, topical anti-inflammatories were tapered over several months, and dorzolamide/timolol was eventually discontinued. On ophthalmic recheck 17 months postoperatively, the patient was visual, comfortable, normotensive and PLR positive OU. There was mild anisocoria, dyscoria and mydriasis OD, aphakia OD, pseudophakia OS, no aqueous flare OU, minimal capsular opacity and an unchanged retinal examination with numerous punctate gray lesions in the dorsal retina OU. Medications consisted of 0.1% Nevanac (OU q24h). At home, vision was reportedly very good.

## Discussion

This case series demonstrates that intralenticular *E cuniculi* is a potential cause of cataracts, uveitis and secondary glaucoma in domestic cats in California, USA.

Although *E cuniculi* is found worldwide, feline ocular encephalitozoonosis has only been reported in Austria,^16^ France^10^ and the USA (feline cornea).^12^ Factors including climate and animal reservoirs may affect *E cuniculi*’s prevalence and risk to cats. Specifically, environmental spore viability varies by temperature.^3^ Given that encephalitozoonosis in rodents has been documented worldwide,^5,17–22^ rodents likely spread disease, as corroborated by case 1 and several Austrian cases that tested positive for the mouse strain (strain II).^16^

Although *E cuniculi* is an opportunistic pathogen in immunocompromised people,^23^ the role of immunosuppression in feline ocular encephalitozoonosis remains unclear. The cases in this study were immunocompromised due to concurrent diseases (see Table 1) and immunomodulatory drugs (prednisolone and chlorambucil in case 2). This aligns with the current understanding that immunosuppression exacerbates rabbit encephalitozoonosis.^4^ However, in the 2011 study published by Benz et al,^16^ 11 systemically healthy European Shorthair cats also developed cataracts and uveitis from *E cuniculi*, though 4/11 cats had positive titers for *Toxoplasma gondii* (IgG 1:4000). In the same study, 2/100 ophthalmologically healthy cats had a positive antibody titer for *E cuniculi*. Research conducted in North America,^24,25^ Europe^18,26,27^ and Asia^28–30^ has found that *E cuniculi* prevalence range from 0% to 26.8%, with one paper demonstrating a seroprevalence of 6.1% (18/295)^30^ in healthy, asymptomatic cats.

The mechanism by which *E cuniculi* causes uveitis is unknown. *E cuniculi* antigens may contribute to the inflammatory response;^31^ this is supported by Nell et al^11^ and cases 1 and 3, which suggest that focal anterior cataracts due to *E cuniculi* may be more inflammatory relative to focal cataracts due to other etiologies. Alternatively, *E cuniculi* may replicate and physically disrupt the lens, leading to lens-induced uveitis.^7^ In case 1, aqueous humor PCR screening failed to show any evidence of other intraocular infections and supported *E cuniculi* being the causative agent for uveitis.

Currently, phacoemulsification surgery, antiparasitic medication and symptomatic treatment are employed to treat intralenticular *E cuniculi* infections. Phacoemulsification treats cataracts, removes microsporidia and minimizes further pathogen replication and intraocular inflammation. Fenbendazole, often prescribed at ranges of 20–50 mg/kg q24h for 3 weeks (extra-label), targets various pathogen stages.^32^ Symptomatic treatment often includes oral and ophthalmic anti-inflammatories to address anterior uveitis. Since systemic immunosuppression facilitates *E cuniculi*,^33^ corticosteroids should be employed at anti-inflammatory doses. The literature and this report suggest that surgical management of intraocular *E cuniculi* via phacoemulsification, especially early phacoemulsification,^34^ can successfully maintain vision and comfort, while medical management alone may more commonly lead to blindness, discomfort and enucleation.^16^

Various diagnostic testing is available for *E cuniculi* (see Table 2). Serology is a non-invasive, low-risk screening tool that is expected to be weakly or strongly positive for *E cuniculi* in cats with intraocular *E cuniculi*; however, PCR positivity of ocular fluid/tissues provides more definitive evidence of intraocular involvement. PCR detection of *E cuniculi* varies based on sample location. In Benz et al, aqueous humor from 10/19 affected cats was PCR positive, whereas lens material was PCR positive in one or both eyes in 11/11 of these cats.^16^ Histopathology with hematoxylin and eosin stains can help guide the diagnosis of *E cuniculi* (see Figure 3a–c); however, the preferred histologic stains for *E cuniculi* spore detection are modified trichrome and Gram stain with light microscopy and calcofluor white stain with ultraviolet light microscopy,^35^ though acid fast trichome can be effective (see Figure 3d);^16^ in case 3, Luna stain was helpful.

**Figure 2:**
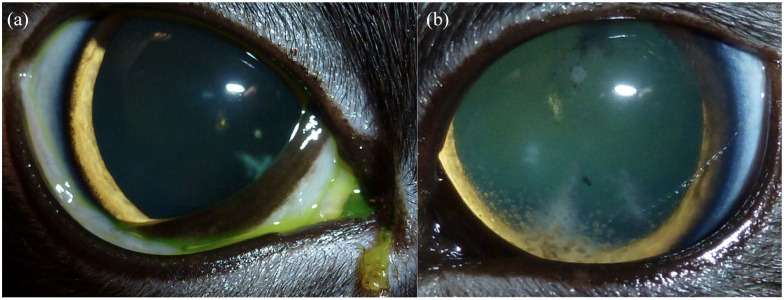
Case 3. Both pupils were dilated with 1% tropicamide (Akorn). (a) OD (initial examination): no aqueous flare, mild keratic precipitates, incipient peripheral cortical cataracts, fluorescein negative. (b) OS (first recheck after 3 weeks): mild iris thickening, trace aqueous humor cells, keratic precipitates, incipient peripheral cortical cataract, fluorescein negative. Images courtesy of Dr Holly Hamilton

When feline cataracts are identified, possible causes include chronic uveitis (most common), trauma (especially penetrating trauma), *E cuniculi*, secondary to glaucoma or lens luxation, congenital, possibly hereditary, nutritional or uncommonly metabolic (hypocalcemia, hyperphosphatemia, diabetes).^36^ The cause of feline cataracts can be very difficult to determine, particularly since chronic uveitis commonly causes cataracts and vice versa. Cataracts caused by chronic lens-induced uveitis and those caused by *E cuniculi* can be very similar in appearance and size (ranging anywhere from incipient to mature in this report). However, in the experience of the authors, *E cuniculi* cataracts typically originate as focal lesions in the anterior cortex and spread from there to the whole lens. As cases are presented at different stages, the appearance of *E cuniculi* cataracts can differ in size and stage of maturity. We suspect that smaller (incipient) *E cuniculi* cataracts can cause disproportionately severe and acute uveitis and/or may progress somewhat more quickly relative to incipient cataracts of other etiologies (with the possible exception of penetrating trauma where an obvious corneal lesion would be expected). In two cases in this report (cases 1 and 3), incipient cataracts were associated with 1/4 or 3/4+ aqueous flare, which is unusual in cataracts not associated with traumatic intralenticular bacterial implantation or long-standing uveitis. However, inflammation caused by *E cuniculi* cataracts can be variable; in case 2, cataracts were immature and uveitis was initially minimal (although this patient was also on oral prednisolone for intestinal lymphoma). Speed of progression of *E cuniculi* cataracts can also be variable. In case 2, cataracts were reported to be rapidly progressive, whereas in case 3 the cataract progressed from incipient to mature over 6 years. Furthermore, features of chronic uveitis that may have led to these cataracts (such as chronic iris discoloration or large areas of posterior synechiation) were generally lacking in the cases presented here, except for mild rubeosis in case 3 and very focal synechiation in case 1 (see Figures 1 and 2). Ultimately, serology for *E cuniculi* is recommended as a screening tool in all cases of feline cataracts for which an alternative underlying cause is not apparent. If *E cuniculi* serology is positive, then referral to an ophthalmologist for additional (PCR) testing of ocular tissues and more intensive medical and surgical treatment should be considered, as this would be expected to improve the clinical outcome.

**Figure 3:**
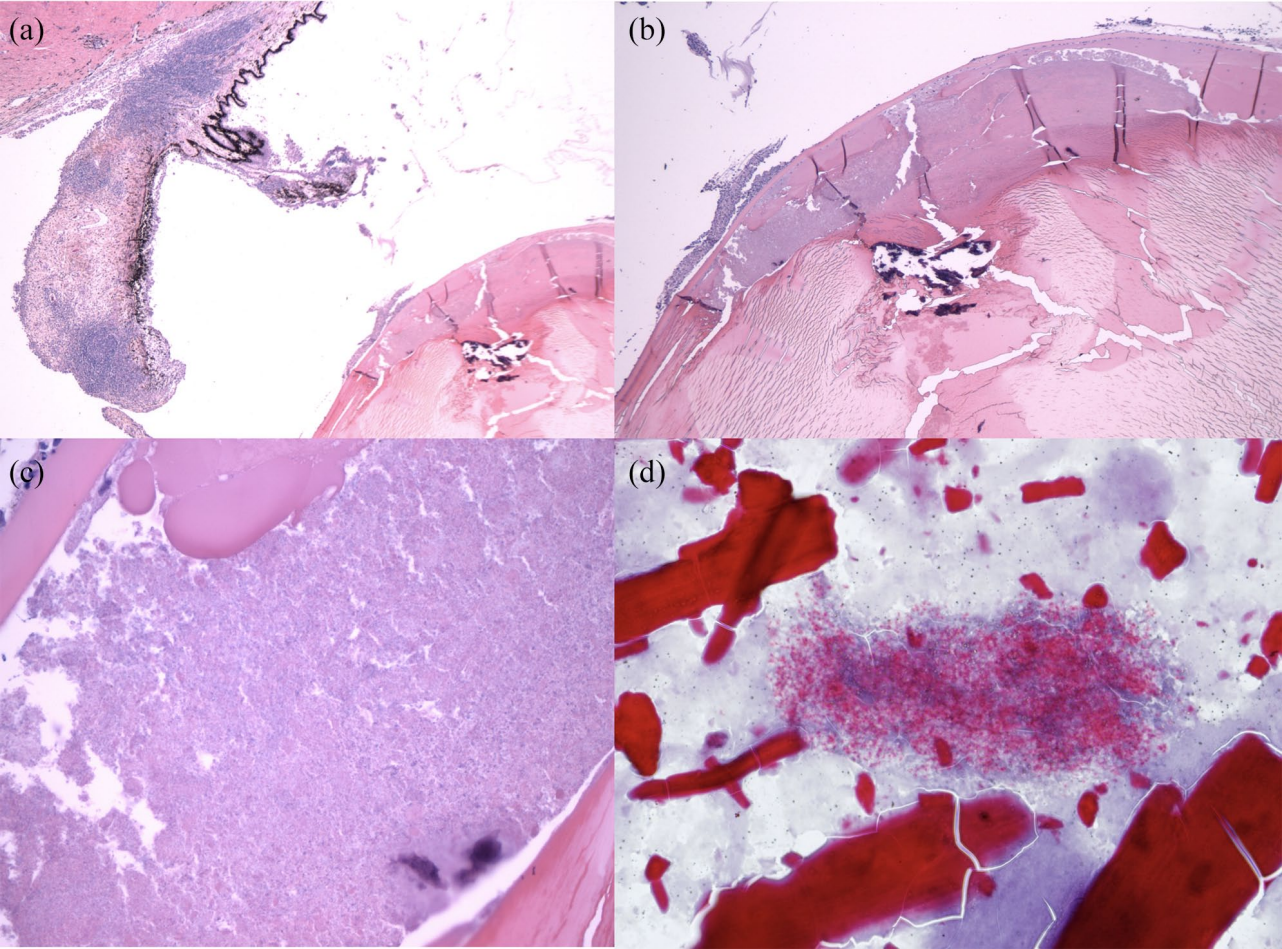
Histopathology. (a) Case 3, hematoxylin and eosin, × 2 magnification showing lymphoplasmacytic iritis. (b) Case 3, hematoxylin and eosin, × 4 magnification showing regionally severe equatorial lens fiber degeneration. (c) Case 3, hematoxylin and eosin, × 40 magnification showing innumerable *Encephalitozoon cuniculi* organisms within the lens, with swollen lens fibers/Morgagnian globules (top center) and a few neutrophils outside the capsule (upper left). (d) Case 1, histopathology. Ziehl–Neelsen acid fast stain, × 60 magnification, lens material and *E cuniculi* organisms. Images (a), (b) and (c) courtesy of Dr Christopher Reilly, DACVP. Image (d) courtesy of Dr Barbara Nell

## Conclusions

This study highlights *E cuniculi* as a cause of feline cataracts in the USA (and likely worldwide). Study limitations include low case numbers and heterogeneous, incomplete patient data with limited follow-up. Although this series provides clinically relevant information, it does not necessarily represent optimal treatment of feline ocular *E cuniculi*. Because the literature on feline encephalitozoonosis is somewhat lacking, future studies should more thoroughly evaluate systemic involvement, pathophysiology and/or best treatment practices. *E cuniculi* should be considered in cats presenting with cataracts, especially those with concurrent anterior uveitis. The authors hope that increased awareness and testing will lead to earlier diagnosis of feline intraocular *E cuniculi* and improved clinical outcomes.
